# Case Report of a Pelvic Kidney with Ureteral Obstruction from Inguinal Hernia Entrapment and Concurrent Cryptorchid Testis

**DOI:** 10.21980/J8F345

**Published:** 2022-04-15

**Authors:** Nathan Feil, Daniel Kwan, Cameron Fateri, Lindsey Spiegelman, Roozbeh Houshyar

**Affiliations:** * University of California, Irvine, Department of Radiological Sciences, Orange, CA; ^ University of California, Irvine, Department of Emergency Medicine, Orange, CA

## Abstract

This is a case report of a ureteral obstruction from inguinal hernia entrapment with an ipsilateral cryptorchid testis in a 45-year-old male with an ectopic pelvic kidney. Ureteral herniation is unusual and to our knowledge has not been described in the setting of a patient with an ectopic pelvic kidney and undescended testis. The patient initially presented at an outside hospital with mild right lower quadrant abdominal pain and scrotal fullness, and initial computed tomography (CT) imaging demonstrated a pelvic right kidney with an indirect right inguinal hernia containing an entrapped ureter resulting in severe hydronephrosis. The CT also revealed an undescended testis on the patient’s right side. A nephrostomy tube was then placed in the right kidney under interventional radiological (IR) guidance and subsequent CT urography showed resolution of hydronephrosis. Surgical hernia repair was followed up on a later date. The discussion addresses the unique pathological features of this case while highlighting the relevant complications of a ureteral herniation.

**Topics:**

Pelvic kidney, renal ectopia, ureteral obstruction, cryptorchidism, undescended testis.

**Figure:**
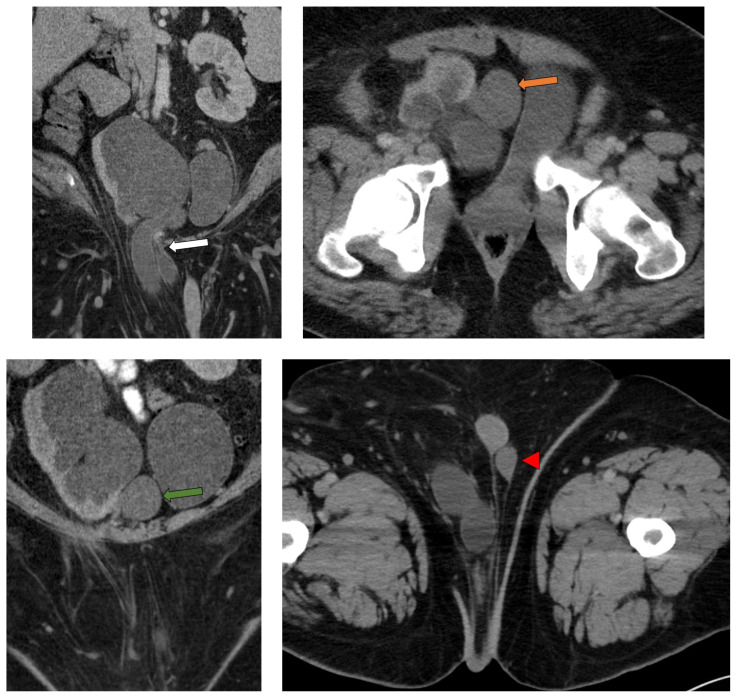


**Figure:**
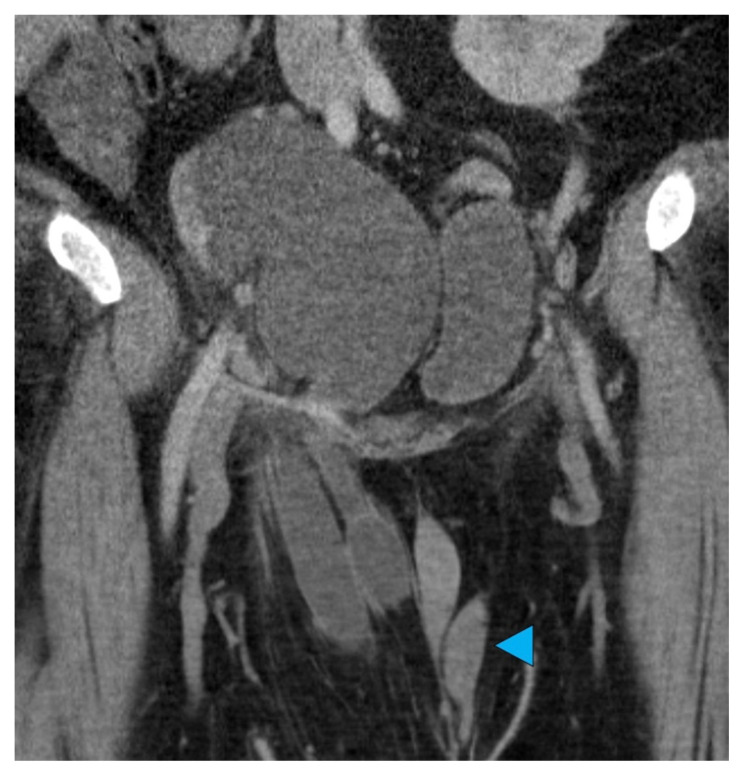


## Brief introduction

Renal ectopia is a product of abnormal kidney migration during early development, with failure of ascent leading to placement of the kidney in the pelvis.^1^ Similarly, undescended testis is the failure of either one or both testes to migrate from the abdomen into the scrotal sac during early development and is of significance given the increased risk for neoplastic transformation. These are relatively uncommon findings in adults, and simultaneous occurrence is even rarer. Here we describe an unusual case of an ectopic pelvic kidney with ureteral obstruction from inguinal hernia entrapment with an ipsilateral cryptorchid testis.

## Presenting concerns and clinical findings

A 45-year-old male with history of obesity, Roux-en-Y gastric bypass, incarcerated hernia with subsequent corrective surgery more than 10 years prior, and a known right inguinal hernia presented to the emergency department at an outside hospital for abnormal outpatient imaging results which had been obtained for increasing right lower quadrant pain and scrotal fullness. On physical examination, the patient was noted to have mild right lower quadrant abdominal tenderness without significant distension.

## Significant findings

The patient was afebrile with normal lactate and white blood cell count. Initial CT imaging showed an ectopic right pelvic kidney with entrapment of his right ureter within an indirect right inguinal hernia causing severe hydronephrosis (coronal: white arrow). Also discovered was an ovoid hypodensity in the right anterior pelvis consistent with right undescended testis (axial: orange arrow; coronal: green arrow) that was previously unknown to the patient, with a normal left scrotal testis (axial: red arrowhead; coronal: blue arrowhead). Other potential etiologies of the patient’s symptoms could include appendicitis or incarcerated inguinal hernia, though the imaging results and absence of systemic inflammatory response syndrome made these causes less likely.

## Patient course

A nephrostomy tube was successfully placed into the right kidney under IR guidance. After initial improvement of the patient’s symptoms, the patient experienced multiple incidents of obstructed drainage due to catheter retraction necessitating replacement. After three such occurrences, the patient presented to the emergency department with suspected pyelonephritis, and CT redemonstrated the ureteral entrapment with a malpositioned anterior approach nephrostomy tube and hydronephrosis. The nephrostomy tube was successfully replaced, antibiotic treatment for pyelonephritis was initiated, and the patient was discharged when stable. The treatment was well-tolerated by the patient and follow-up imaging showed resolution of the hydronephrosis. However, there was persistent marked narrowing of the right ureter through the indirect right inguinal hernia consistent with ureteral entrapment. The patient followed up with general surgery who planned for an elective repair of the right inguinal hernia, and urology consultation was planned for discussion of surgical correction of renal and gonadal issues.

## Discussion

Renal ectopia is a product of abnormal kidney migration during early development, with failure of ascent leading to placement in the pelvis, and the incidence by autopsy is 1/1,000.^1^,^2^ Other than an increased risk for developing hydronephrosis and symptomatic renal calculi due to the tortuous ureter, this variant anatomy is generally thought to have little increased risk for other diseases of the kidney and its related structures.^3^ Ureteral herniation is a rare occurrence, and previous reports of this malady have been described in such instances as an orthotopically transplanted kidney with obstruction of the ureter in an inguinal hernia, bilateral inguinoscrotal herniation, herniation with obstruction from a contralateral pelvic tumor, herniation with obstruction through the superior lumbar triangle, ureterosciatic herniation with renal pelvic rupture, and even the entire kidney into an inguinal hernia.^4^–^9^ To the knowledge of the authors, ureteral entrapment from a congenital ectopic pelvic kidney into an inguinal hernia with ipsilateral cryptorchid testis has not been described.

Although ectopic kidneys (0.4%) have been reported to be associated with cryptorchid testes in children, there is little data for this same relationship in adults.^11^ This is due to the low incidence of cryptorchid testis in patients older than 1 year of age because most cases are discovered shortly after birth. Given that undescended testicles are already a rare finding in post-pubertal men (to the point where incidence has not been determined), it is an even more rare finding to have an associated ectopic kidney. Descent is believed to be hormonally driven (testosterone, estrogen, human chorionic gonadotropin, and insulin-like 3), and a disturbance of normal levels could lead to disruption of the descent process.^12^ Literature is still lacking in the exact process of testicular descent and it is therefore difficult to provide the mechanism for how these anomalies occur. However, the urogenital ridge is the common embryologic origin of the kidneys (metanephros) and gonads (mesonephros) and makes such associations likely. Indeed, a case of pelvic kidney with ipsilateral undescended testis has been reported previously, though without the complications of herniation as described in the present case.^13^

This case demonstrates a rare complication of a rare phenomenon that is important for medical providers to recognize and understand. The tortuous nature of the ureter in ectopic kidneys makes them more susceptible to the complications of hydronephrosis, and perhaps more susceptible to a herniation that could potentially lead to further obstruction. The resultant obstructive uropathy may lead to acute kidney injury, urinary tract infection, septicemia, and even chronic kidney disease or renal failure.^14^ Early CT imaging, as was obtained in this case, is important in making this diagnosis and ruling out other life-threatening causes of abdominal pain. Consultation for management of the hernia by a general surgeon and renal and testicular pathology by a urologist are necessary for long-term management.

## Supplementary Information








