# Supplementary figures and images for: Case Report of a Pelvic Kidney with Ureteral Obstruction from Inguinal Hernia Entrapment and Concurrent Cryptorchid Testis
Source: J Educ Teach Emerg Med. 2022 Apr 15;7(2):V28–32. doi: 10.21980/J8F345 (PMC10332742; doi:10.21980/J8F345)

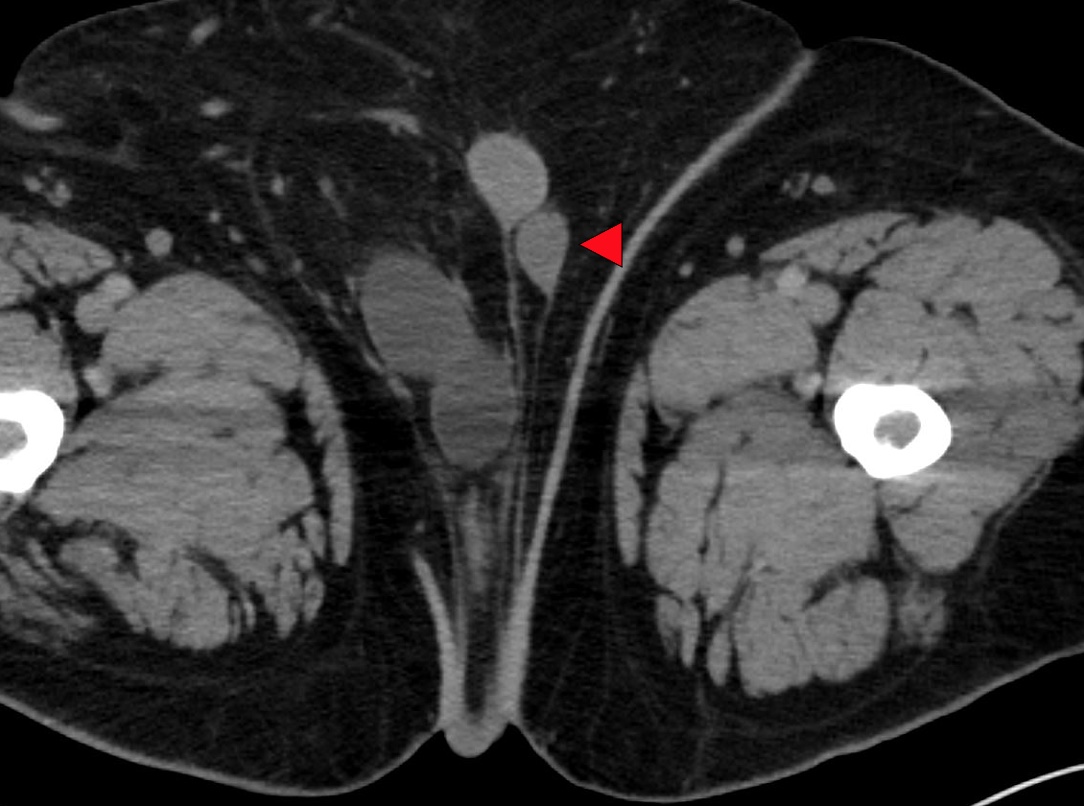

Supplement: Supplementary file 1 [file JETem-7-2-V28-supp1.jpg]

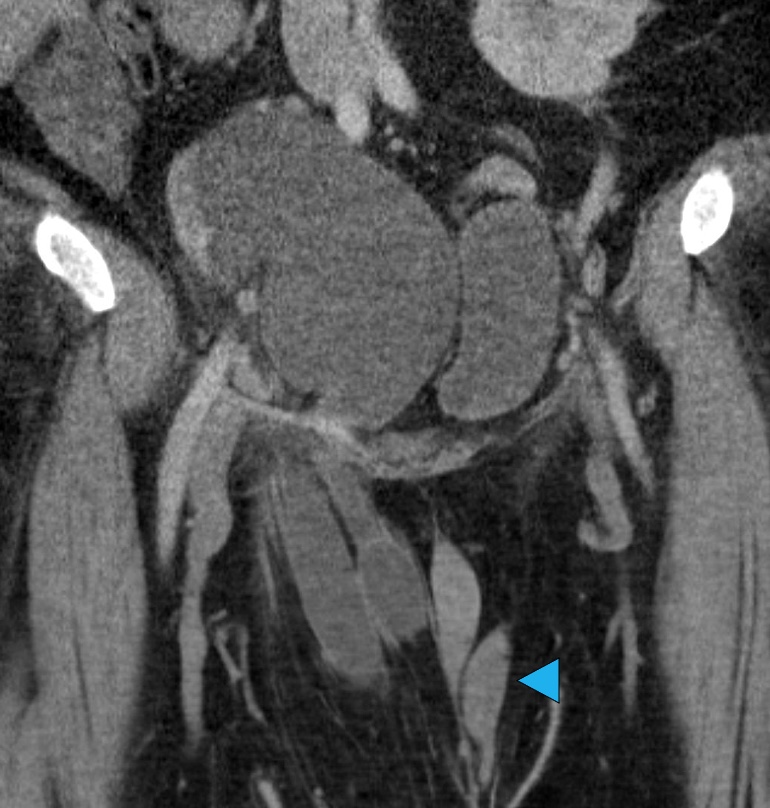

Supplement: Supplementary file 2 [file JETem-7-2-V28-supp2.jpg]

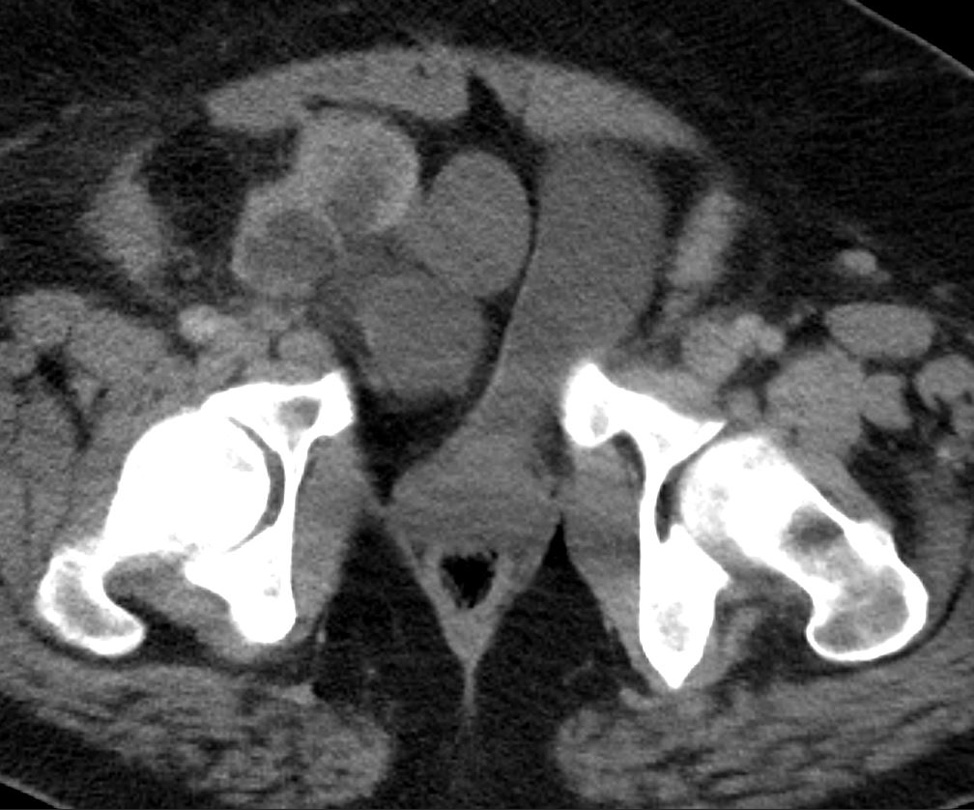

Supplement: Supplementary file 3 [file JETem-7-2-V28-supp3.jpg]

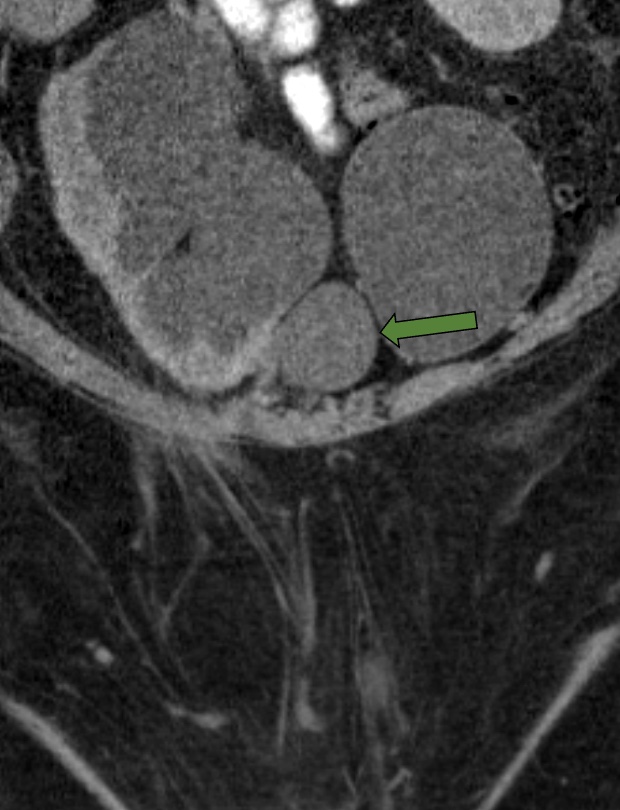

Supplement: Supplementary file 4 [file JETem-7-2-V28-supp4.jpg]
